# Lymphedema self-care: economic cost savings and opportunities to improve adherence

**DOI:** 10.1186/s12962-023-00455-7

**Published:** 2023-07-29

**Authors:** Pinar Karaca-Mandic, Craig A. Solid, Jane M. Armer, Roman Skoracki, Elizabeth Campione, Stanley G. Rockson

**Affiliations:** 1 Carlson School of Management, University of Minnesota, Carlson School of Management, 321 19th Avenue South, Minneapolis, MN 55455 USA; 2 Solid Research Group, St Paul, Minneapolis, MN USA; 3 University of Missouri Sinclair School of Nursing, Columbia, MO USA; 4 James Cancer Treatment and Research Center, Ohio State University, Columbus, OH USA; 5 Physical Therapy Program, Midwestern University, Downers Grove, IL USA; 6 Stanford Center for Lymphatic and Venous Disorders, Division of Cardiovascular Medicine, Stanford University School of Medicine, Stanford, CA USA

**Keywords:** Breast cancer-related lymphedema, Economic burden, Cancer survivors, Quality of life, Self-care, Economic benefit, Economic burden, Complications, Pneumatic compression device

## Abstract

**Background:**

Breast cancer-related lymphedema (BCRL) imposes a significant economic burden on patients, providers, and society. There is no curative therapy for BCRL, but management through self-care can reduce symptoms and lower the risk of adverse events.

**Main body:**

The economic burden of BCRL stems from related adverse events, reductions in productivity and employment, and the burden placed on non-medical caregivers. Self-care regimens often include manual lymphatic drainage, compression garments, and meticulous skin care, and may incorporate pneumatic compression devices. These regimens can be effective in managing BCRL, but patients cite inconvenience and interference with daily activities as potential barriers to self-care adherence. As a result, adherence is generally poor and often worsens with time. Because self-care is on-going, poor adherence reduces the effectiveness of regimens and leads to costly treatment of BCRL complications.

**Conclusion:**

Novel self-care solutions that are more convenient and that interfere less with daily activities could increase self-care adherence and ultimately reduce complication-related costs of BCRL.

## Background

### Economic impact of BCRL

As many as 40% of breast cancer survivors may develop breast cancer-related lymphedema (BCRL) [1] where protein-rich fluid accumulates in the ipsilateral upper extremity, causing swelling, pain, and fatigue. In 2021, De Vrieze et al. published an analysis of the costs associated with treating BCRL in Belgium, [2] less than 2 years after an overlapping group of authors published a review of eight studies that assessed the financial burden of BCRL on patients and society [3]. The conclusions of both were the same: BCRL imposes a significant economic burden upon patients, payers, and society. The authors reported €2249 ($2449 in 2021 USD) and up to USD$3165 in average direct healthcare costs for a year of decongestive lymphatic treatment (DLT), including up to USD$2574 by patients; indirect costs to patients (e.g., lost wages, reduced productivity, etc.) could be as high as USD$5545 per year [2, 3]. In one of the studies reviewed by the authors, 2.3% of more than 56,000 lumpectomy/mastectomy-treated patients experienced at least one hospitalization for complicated lymphedema within 2 years, with these patients incurring an additional $26,269 in healthcare costs during that time period [4]. Others have also demonstrated the significant economic burden of BCRL. For example, in a matched cohort analysis of working-age women with breast cancer, patients with BCRL incurred $8,290 more costs, on average, during the first 2 years of cancer treatment, as compared to survivors without BCRL [5]. Notably, De Vrieze and colleagues suggest that some studies likely underestimate costs or include an incomplete assessment of total costs incurred, [3] and that, often, indirect costs related to variables like transportation and loss of productivity, which can be substantial, are often omitted [2].

As an example of the indirect costs, more than two in five (42%) individuals with BCRL report that lymphedema has negatively impacted their work performance; among those with severe lymphedema, the percentage is 75% [6]. Additionally, those with lymphedema leave the workforce more often than those without. In a study of breast cancer patients by Bulley et al., the rate of work stoppage was more than twice as high in lymphedema patients (15.5% versus 6.1%) [7]. Indirect costs can also stem from caregiver burden. When severe, lymphedema requires significant time and effort from non-medical caregivers; as arm disability, pain, grip strength, and lymphedema duration increase, so does the burden on caregivers [8].

In this commentary we aim to explore how poor adherence to self-care contributes to this cost, the barriers associated with self-care, and how novel self-care solutions could reduce BCRL-related costs by improving patient adherence to reduce the risk of BCRL complications.

## Main text

### Treatment of BCRL and related cost avoidance

The current “gold standard” of care for lymphedema involves complex decongestive therapy (CDT), consisting of manual lymph drainage (MLD), the use of compression bandaging and garments, meticulous skin care, and remedial exercise [9]. CDT is composed of an intensive phase under the direct care of trained lymphedema therapists, followed by the maintenance self-care phase carried out by the person with BCRL. Long-term self-care is necessary to maintain limb health and avoid related complications. In addition to CDT, pneumatic compression devices (PCDs) can be used as a self-care strategy to help reduce limb volume and improve outcomes.

The use of PCDs has been linked to significantly lower costs and lower utilization in this population. Specifically, with use of PCDs, studies have reported reductions in hospitalizations, outpatient visits, urgent care visits, documented episodes of cellulitis, and utilization of physical therapy resources [10–12]. For example, in a study of 374 cancer patients (76% breast cancer survivors), the adjusted rate of outpatient hospitalizations dropped from 58.6 to 41.4% after treatment with a PCD (p < 0.001); and total adjusted outpatient lymphedema-related costs dropped from USD$1517 to USD$694 (p < 0.001) [12]. A retrospective claims analysis of over 1,000 cancer-related lymphedema patients observed that PCD use was associated with reductions in hospitalization rates from 45 to 32% (p < 0.0001), outpatient hospital visits from 95 to 90% (p < 0.0001), and physical therapy use from 50 to 41% (p < 0.0001); average healthcare costs dropped more than $11,000 per patient (p < 0.0001) [10]. These reflect reductions in yearly healthcare costs of 22% to 37% in the year after acquiring a PCD [10, 12]. Notably, these results are not unique to BCRL. A study of secondary lymphedema more generally and the impact of pneumatic compression on lower extremity lymphedema at a single health center reported a reduction in the average number of hospitalizations for lymphedema-associated complications from 0.84 to 0.16 per patient per year, resulting in $3200 in savings per patient [11].

### Barriers to adherence to self-care

Unfortunately, overall adherence to self-care (use of compression garments, use of PCDs, etc.) is poor [13–15] and declines with the length of time since lymphedema diagnosis and with edema severity [16, 17]. For example, when 141 breast cancer survivors were asked about various BCRL self-care modalities, only 60% of individuals with compression garment prescriptions actually wore the garments; and while 72.5% reported adhering to skin care regiments at least 75% of the time, only 30% reported that same level of adherence for bandaging, lymphatic drainage, and PCD use [13]. The reasons for non-adherence are numerous. Patients have cited time constraints, discomfort, and the inconvenience of treatments as barriers to self-care adherence [15, 18]. Additionally, when patients feel that self-care treatments interfere with daily activities, they are less likely to be adherent [19]. There are long-standing concerns that the discomfort and inconvenience of compression garments may reduce adherence to their use [20]. A review of the lymphedema-related literature identified several barriers to self-care adherence, including the complexity of treatment regimens, symptom burden, and a lack of education and support [14].

These self-care barriers mirror those cited in the literature that relate to the general problem of chronic disease self-management. Studies of a variety of chronic diseases suggest that when patients believe self-management tasks are time-consuming, inconvenient, complex, or burdensome, they are less adherent to self-care [21]. For those who self-manage lymphedema after the intensive treatment phase conducted by professional lymphedema therapists, treatment adherence is an important factor for treatment success. One study reported that treatment failure rates (defined as lymphedema volume increase (LVI) of at least 50% of the total reduction obtained during the intensive phase) was 38% at 1 year, 53% at 2 years, and 65% at 4 years. More importantly, non-compliance with the use of compression garments was associated with the likelihood of treatment failure [22].

### A novel self-care solution as an opportunity to improve adherence

Clinical and economic research clearly demonstrates that lymphedema imposes a significant burden on patients and the healthcare system. Self-management is a life-long commitment, and key to managing limb health and avoiding complications. Pneumatic compression as an adjunct to CDT may be an important component of self-care, but adherence has been shown to be poor. Technologies that reduce disruptions to daily life may increase adherence to self-care that subsequently result in improvements in patient outcomes. Medical device and medical technology companies seek to improve patient care through the development of innovative solutions that confer both a clinical and economic benefit. Recently, Koya Medical, Inc. (a company the authors have helped to advise) developed and tested a novel non-pneumatic compression device (NPCD) against a traditional PCD in a randomized cross-over trial [23]. The NPCD was designed to incorporate patient mobility, so that patients could remain active during compression therapy. Study subjects overwhelmingly preferred the NPCD, were significantly more adherent to its use, and confirmed that it allowed them to remain active and even exercise while wearing the NPCD. Quality-of-life metrics improved with the NPCD, while they remained static with the PCD. Clinically, subjects achieved significantly greater reductions in limb edema with the NPCD than they did with the PCD. In short, the NPCD produced better clinical and quality-of-life outcomes with better adherence and patient satisfaction. Innovations, such as the NPCD, that incorporate mobility can serve as an important opportunity to increase adherence to self-care. This type of innovative solution supports the patient, reduces complication-related health encounters, as well as costs, among lymphedema patients, and ultimately improves outcomes.

## Conclusions

Patient-centered innovations for individuals with BCRL can improve adherence to self-care and reduce complications and costly healthcare utilization. The pursuit of additional novel solutions to support self-care may confer both clinical improvements and economic savings. As evidenced by the novel NPCD, such solutions can ultimately reduce costs by improving patients’ ability to manage their BCRL.

## Data Availability

Not applicable.

## Abbreviations

BCRL: Breast cancer-related lymphedema
CDT: Complex decongestive therapy
LVI: Lymphedema volume increase
MLD: Manual lymph drainage
NPCD: Non-pneumatic compression device
PCD: Pneumatic compression devices
